# Emodin attenuates CFA-induced inflammatory pain in association with suppression of the Cx3cl1/Cx3cr1/p38 MAPK signaling axis

**DOI:** 10.3389/fphar.2026.1953662

**Published:** 2026-10-14

**Authors:** Xiang Zheng, Jiabao Dai, Changyuan Mao, Weidong Zhang, Jing Huang, Shuqin Hu

**Affiliations:** 1 Department of Pain Medicine, The Second People’s Hospital of Changzhou, the Third Affiliated Hospital of Nanjing Medical University, Changzhou, Jiangsu, China; 2 Department of Anesthesiology, The Second People’s Hospital of Changzhou, the Third Affiliated Hospital of Nanjing Medical University, Changzhou, Jiangsu, China; 3 Department of Pediatrics, The Second People’s Hospital of Changzhou, the Third Affiliated Hospital of Nanjing Medical University, Changzhou, Jiangsu, China

**Keywords:** analgesia, complete freund’s adjuvant (CFA), Cx3c1/Cx3cr1, emodin, inflammatory cytokines, inflammatory pain, p38 MAPK

## Abstract

**Objectives:**

To investigate the analgesic effect of emodin on Complete Freund’s Adjuvant (CFA)-induced inflammatory pain and to clarify the underlying molecular mechanism.

**Methods:**

Emodin was administered intraperitoneally or prophylactically to attenuate CFA-induced inflammatory pain. RNA-seq was performed to identify the biological functions of differentially expressed genes in mouse dorsal root ganglion (DRG) tissues. The expression levels of Cx3cl1/Cx3cr1 were evaluated by RT-qPCR, Western blotting, and immunofluorescence. Pharmacological and genetic approaches were employed to block Cx3cr1 and assess its effect on pain thresholds in CFA-treated mice. Hematoxylin and eosin (H&E) staining was used to examine the infiltration of immune and inflammatory cells in dorsal root ganglion (DRG) tissues before and after emodin treatment. Following emodin treatment, the levels of p-p38/p38 in DRG tissues were determined by Western blotting, and the mRNA levels of the pro-inflammatory cytokines *Tnf-α*, *Il-1β*, and *Il-6* in DRG tissues were measured by RT-qPCR.

**Results:**

Both therapeutic and prophylactic administration of emodin effectively alleviated CFA-induced mechanical and thermal hypersensitivity. Emodin treatment suppressed the CFA-induced upregulation of Cx3cl1/Cx3cr1 mRNA and protein levels in the DRG. Pharmacological and genetic blockade of Cx3cr1 effectively attenuated CFA-induced hyperalgesia. In addition, emodin markedly inhibited p-p38/p38 protein expression in the DRG of mice with CFA-induced inflammatory pain, reduced immune cell infiltration in DRG tissues, and downregulated the levels of the pro-inflammatory cytokines *Tnf-α*, *Il-1β*, and *Il-6*.

**Conclusion:**

Emodin attenuated CFA-induced inflammatory pain in mice, an effect associated with reduced Cx3cl1/Cx3cr1 signaling, decreased p38 MAPK activation, diminished immune-cell infiltration in the DRG, and lower expression of the proinflammatory cytokines *Tnf-α*, *Il-1β*, and *Il-6* in DRG tissue.

## 1 Introduction

Pain is one of the most common clinical and daily health problems, and inflammatory pain is a major subtype (Toikumo et al., 2025). Inflammatory pain is closely associated with inflammatory lesions. Nociceptors in the trigeminal ganglia and dorsal root ganglia transmit nociceptive signals to the brain upon detection of noxious chemical, thermal, and mechanical stimuli, ultimately leading to pain perception (Bortsov et al., 2025; Grace and Watkins, 2025). During inflammation, the organism exhibits enhanced responses to noxious stimuli (hyperalgesia) or pain evoked by normally innocuous stimuli (allodynia) (Tsagareli, 2019). If left untreated, inflammatory pain may progress to chronic pain, in which pain persists for a prolonged period even after the initial inflammatory insult has been resolved (Jiang et al., 2025). It is well recognized that inflammatory pain severely impairs patients’ quality of life and work capacity, and progression from inflammatory pain to chronic pain imposes a substantial economic burden on both families and society (Hallberg et al., 2023).

It has been reported that physical injuries such as mechanical trauma and high fever, together with neurochemical mediators and inflammatory factors in the nervous system, may trigger inflammatory pain; however, the underlying mechanisms have not yet been fully elucidated (Bruehl, 2015; Cohen and Mao, 2014). In addition, due to the limited progress in current research and therapeutic strategies, the clinical management of inflammatory pain remains a major challenge (Baker and Perazella, 2020).

Emodin (1,3,8-trihydroxy-6-methylanthraquinone) is a naturally occurring anthraquinone derivative and a major bioactive constituent of Rheum palmatum and several medicinal herbs from the Rutaceae, Rhamnaceae, and Leguminosae families (Semwal et al., 2021). Accumulating evidence has confirmed a variety of biological activities of emodin, including antiviral, antibacterial, anti-inflammatory, immunosuppressive, and pro-apoptotic effects (Fu et al., 2025; Shao et al., 2022; Tian et al., 2025; Xia et al., 2019). Importantly, rhubarb has long been widely used in traditional Chinese medicine (TCM) for the clinical treatment of constipation and pain-related disorders (Dong et al., 2016). To characterize the functional role of emodin in inflammatory pain, this study used a Complete Freund’s Adjuvant (CFA)-induced inflammatory pain mouse model to evaluate its analgesic effects and further elucidate the underlying molecular mechanisms involved in pain relief.

## 2 Materials and methods

### 2.1 Animals and drug administration

All animal experiments were performed in accordance with the guidelines approved by the Animal Care and Welfare Committee of Changzhou Second People’s Hospital Affiliated to Nanjing Medical University. Healthy male C57BL/6 mice aged 8 weeks with body weight of 25 ± 1 g were selected for pain behavioral assays and establishment of inflammatory pain models. Mice were housed in temperature-controlled mini-cages at 25 °C ± 1 °C with relative humidity maintained at 55% ± 5% under a 12 h light/dark cycle. Standard chow and sterile distilled water were provided *ad libitum*.

Baseline nociceptive thresholds were measured 1 day before model establishment. Inflammatory pain was induced in mice as described previously by subcutaneously injecting 20 μL of complete Freund’s adjuvant (CFA; F5881, Sigma-Aldrich) into the dorsal surface of the right hind paw (Wang et al., 2025). Mice in the control group received an equal volume (20 μL) of sterile saline at the same site. Mechanical allodynia and thermal hyperalgesia were assessed at 1, 3, 5, 7, 10, and 14 days after CFA injection. Emodin (HY-14393, MedChemExpress) was dissolved and administered intraperitoneally to CFA-treated mice at graded doses of 0, 5, 25, 50, or 100 mg/kg (n = 6–8 mice per group).

### 2.2 Pain behavioral tests

Mechanical pain sensitivity was measured using von Frey monofilaments by recording paw withdrawal reflexes in response to progressively increased filament force. Briefly, mice were placed in plastic cages with wire mesh bottoms and allowed to acclimate for 15–30 min before testing. Von Frey filaments (force range: 0.008–2 g, starting at 0.008 g) were vertically applied to the mid-plantar region of the right hind paw for 6–8 s until the filament bent. Paw withdrawal thresholds (PWTs) were recorded in grams as the readout value (Wei et al., 2021). A hot plate test was adopted to measure paw retraction latency (PWL) for evaluation of thermal hyperalgesia.

The hot plate apparatus consisted of a 25 cm × 25 cm metal heating plate covered with a transparent glass cage. Mice were habituated on the unheated hot plate for 10 min prior to formal testing. The hot-plate test was conducted at 55 °C, with a maximum exposure time of 30 s to minimize the risk of tissue injury. The temperature was selected according to the established experimental protocol used for evaluating thermal nociception in inflammatory pain models. The latency to the first clearly observed nocifensive response, defined as hind-paw licking, hind-paw withdrawal or lifting, jumping, or an escape-oriented movement, was recorded. If no response occurred, the trial was terminated at 30 s, which was treated as the maximum cutoff value rather than as a nocifensive response (Wu et al., 2024).

Animals were randomly assigned to the experimental groups using a computer-generated randomization sequence. Each animal was identified by a coded number, and the experimenter conducting the von Frey and hot plate tests was blinded to the treatment allocation. Behavioral assessments were performed in a predetermined randomized order. The treatment codes were revealed only after completion of the behavioral testing, data recording, and initial analysis.

### 2.3 Transcriptome sequencing and bioinformatics analysis

Transcriptome sequencing was performed on L4–L6 DRG tissues isolated from mice with CFA-induced inflammatory pain. DRG tissues from the Sham and CFA-induced inflammatory pain groups were submitted to Beijing Tsingke Biotech Co., Ltd. (Beijing, China) for transcriptome sequencing and bioinformatics analysis. Differential expression analysis of DRG tissues from the CFA and Sham groups was performed using the DESeq2 R package (v1.26.0). The resulting *P* values were adjusted using the Benjamini-Hochberg method to control the false discovery rate (FDR). Genes with an FDR <0.05 and |log2 (fold change)| ≥ 1 as determined by DESeq2 were defined as differentially expressed genes (DEGs) (Roy et al., 2026). For heatmap visualization, expression values were log2-transformed and then row-scaled using Z-score normalization to facilitate comparison across samples. Hierarchical clustering was performed using the Euclidean distance metric and complete linkage method. Heatmaps were generated using the pheatmap package in R to display the expression patterns of DEGs among the different experimental groups (Wang et al., 2023). Gene Ontology (GO) enrichment analysis of DEGs was conducted using the GOseq R package, which is based on the Wallenius noncentral hypergeometric distribution and corrects for gene length bias among DEGs (Li et al., 2021). KEGG is a database resource used to understand higher-level functions and utilities of biological systems, such as cells, organisms, and ecosystems, from molecular-level information, particularly large-scale molecular datasets generated by genome sequencing and other high-throughput experimental techniques (http://www.genome.jp/kegg/) (Kanehisa et al., 2023). We used KOBAS software to test the statistical enrichment of DEGs in KEGG pathways (Wu et al., 2021).

### 2.4 Reverse transcription quantitative PCR (qRT-PCR)

DRG tissues were harvested from mice. Total RNA was extracted from each sample using TRIzol reagent (Invitrogen, United States) per the manufacturer’s instructions. RNA concentration was quantified with a Nanodrop 2000 spectrophotometer. A total of 1 μg RNA per sample was reverse-transcribed into complementary DNA (cDNA) using the PrimerScript RT Master Mix kit (Takara Bio, Japan). The qPCR reaction mixture was prepared in line with the instructions of SYBR® Premix Ex Taq™ kit (Takara Bio, Japan). The amplification program was set as: initial denaturation at 95 °C for 3 min, followed by 40 cycles of 95 °C for 10 s and 58 °C for 10 s.

Primer sequences used in qPCR were listed below: *Cx3cl1*: Forward 5′-GCG​CTT​TTC​CAG​GAG​AGG-3′, Reverse 5′-GCT​CTG​GTA​AGC​AAA​CAT​GG-3′; *Cx3cr1*: Forward 5′-TCA​CTG​ACA​TCT​ACC​TCC​TG-3′, Reverse 5′- GAT​GCG​GAA​GTA​GCA​AAA​GC-3′; *Tnf*-α: Forward 5′-CAG​GCG​GTG​CCT​ATG​TCT​C-3′, Reverse 5′-CGA​TCA​CCC​CGA​AGT​TCA​GTA​G-3′; *IL-1*β: Forward 5′-CAA​CCA​ACA​AGT​GAT​ATT​CTC​CAT​G-3′, Reverse 5′-GAT​CCA​CAC​TCT​CCA​GCT​GCA-3′; *IL-6*: Forward 5′-CCA​AGA​GGT​GAG​TGC​TTC​CC-3′, Reverse 5′-CTG​TTG​TTC​AGA​CTC​TCT​CCC​T-3′; *Gapdh*: Forward 5′-AGG​TCG​GTG​TGA​ACG​GAT​TTG-3′, Reverse 5′-GGG​GTC​GTT​GAT​GGC​AAC​A-3′. Relative gene expression levels were calculated via the 2^−ΔΔCq^ method using quantification cycle (Cq) values, with *Gapdh* servedas the internal reference gene.

### 2.5 Western blot analysis

To determine the expression levels of Cx3cl1, Cx3cr1, p-p38, p38, GAPDH, and Actin in DRG tissues from CFA-induced inflammatory pain mice, DRG samples were rapidly collected at the indicated time points and homogenized in ice-cold RIPA lysis buffer supplemented with protease and phosphatase inhibitors. The lysates were incubated on ice for 30 min and then centrifuged at 12,000 g for 15 min at 4 °C to obtain the supernatants. Protein concentrations were measured using a BCA protein assay kit. Equal amounts of protein were separated by SDS-PAGE and subsequently transferred onto polyvinylidene fluoride (PVDF) membranes. After blocking with 5% non-fat milk or bovine serum albumin for 1 h at room temperature, the membranes were incubated overnight at 4 °C with primary antibodies against Cx3cl1 (10108-2-AP, Proteintech, IL, Unites States ), Cx3cr1 (29819-1-AP, Proteintech, IL, Unites States), p-p38 (#4511, Cell Signaling Technology, MA, Unites States), p38 MAPK (14064-1-AP, Proteintech, IL, Unites States), GAPDH (10494-1-AP, Proteintech, IL, Unites States), and Actin (20536-1-AP, Proteintech, IL, Unites States). Following washing, the membranes were incubated with appropriate horseradish peroxidase-conjugated secondary antibodies for 1 h at room temperature. Protein bands were visualized using an enhanced chemiluminescence detection system and quantified by densitometric analysis using ImageJ software. The relative expression levels of target proteins were normalized to GAPDH or Actin, and the ratio of p-p38 to total p38 was calculated for phosphorylation analysis.

### 2.6 Cryosectioning and immunofluorescence staining of DRG tissues

To examine the expression of Cx3cr1 in the DRG of CFA-induced inflammatory pain mice, DRG tissues were harvested at the indicated time points after treatment and fixed in 4% paraformaldehyde overnight at 4 °C. The tissues were then cryoprotected in 20% and subsequently 30% sucrose solution until they sank, embedded in optimal cutting temperature (OCT) compound, and sectioned into 10 μm thick frozen sections using a cryostat. The sections were mounted on glass slides, air-dried, and washed with PBS. After permeabilization with 0.3% Triton X-100 and blocking with 5% bovine serum albumin or normal serum, the sections were incubated overnight at 4 °C with the primary antibody against Cx3cr1. After washing, the sections were incubated with the corresponding fluorescent secondary antibody in the dark at room temperature. Finally, the slides were mounted with antifade medium and examined under a fluorescence or confocal microscope. Representative images were acquired, and fluorescence intensity was quantified using ImageJ.

### 2.7 Molecular docking

The 2D structure of emodin was obtained from the PubChem database, imported into Chem3D 23.1.1, and used to generate and optimize the 3D structure, which was then saved in MOL2 format. The crystal structure of the Cx3cr1 protein target was retrieved from the RCSB PDB database by selecting a high-resolution structure (Burley et al., 2023). Water molecules, phosphate groups, and other redundant moieties were removed using PyMOL 2.6, and the preprocessed structure was saved in PDB format (Bramucci et al., 2012). AutoDock Tools was used to add hydrogens, remove water, assign charges, and define rotatable bonds for Cx3cr1 and emodin (Morris et al., 2009)]. Molecular docking simulations were then performed using AutoDock Vina 1.2.5 (Trott and Olson, 2010). Docking box coordinates and dimensions were defined according to the active pocket of the protein, and the optimal binding conformation was selected based on binding free energy scoring. Interaction visualization was performed using Discovery Studio 2019 and PyMOL 2.6 to generate 2D interaction maps and 3D binding mode figures (Bramucci et al., 2012).

### 2.8 HE staining of DRG tissues

To evaluate histopathological changes in the dorsal root ganglion (DRG) following CFA-induced inflammatory pain, DRG tissues were collected from mice after emodin treatment. The harvested tissues were fixed in 4% paraformaldehyde overnight, dehydrated through a graded ethanol series, cleared in xylene, and embedded in paraffin. Paraffin blocks were sectioned into 5 μm thick slices and mounted on glass slides. After deparaffinization and rehydration, the sections were stained with hematoxylin, differentiated, and counterstained with eosin according to standard protocols. Subsequently, the sections were dehydrated, cleared, and mounted with a neutral resin medium. Histological images were captured using a light microscope, and inflammatory cell infiltration and morphological alterations in the DRG were evaluated under blinded conditions.

### 2.9 Data collection and statistical analysis

All experimental data were obtained from at least three independent biological replicates and are presented as the mean ± standard deviation (SD). Quantitative comparisons among groups were performed using one-way or two-way repeated-measures analysis of variance (ANOVA) followed by Sidak multiple-comparisons test, as appropriate. A *P* value of less than 0.05 was considered statistically significant. Statistical analyses and graph generation were performed using GraphPad Prism 8 and SPSS 22.0 software.

## 3 Results

### 3.1 Establishment of the CFA-induced inflammatory pain mouse model

To establish a mouse model of inflammatory pain, 8-week-old C57BL/6 mice were subcutaneously injected with 20 μL of CFA into the dorsal hind paw. Several hours after injection, clear local inflammatory signs, including reddish-purple swelling, were observed at the injection site. As reported previously (Ao et al., 2025), these manifestations confirmed the ability of CFA to induce inflammatory pain. As shown in Figure 1, compared with the saline control group, the CFA-treated group exhibited a significant reduction in pain thresholds for both mechanical sensitivity (Figure 1B) and thermal sensitivity (Figure 1C) on day 1 after CFA injection (P < 0.001), and this decrease persisted until day 14. Collectively, these results validated the successful establishment of a CFA-induced inflammatory pain mouse model.

**FIGURE 1:**
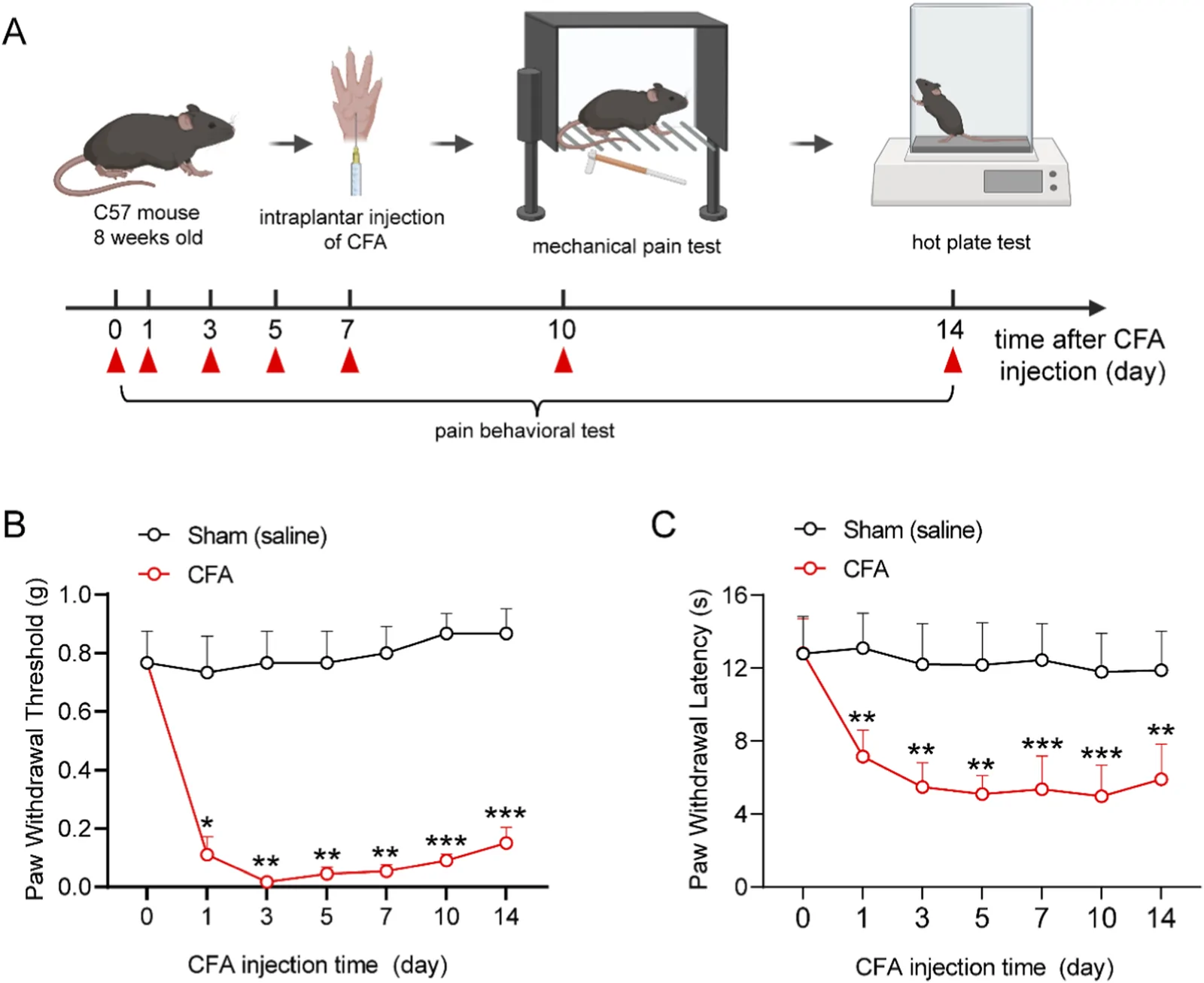
CFA-induced inflammatory pain model in mice. **(A)** Schematic illustration of CFA-induced inflammatory pain in mice; **(B)** von Frey test for assessing the mechanical pain threshold in the CFA-induced inflammatory pain mouse model; **(C)** hot plate test for measuring the thermal hyperalgesia threshold in the CFA-induced inflammatory pain mouse model. All data are presented as mean ± SEM. Statistical analysis was performed using two-way repeated-measures analysis of variance (ANOVA) followed by Sidak multiple-comparisons test. *P < 0.05, **P < 0.01 and ***P < 0.001 indicates a statistically significant difference in pain thresholds between the CFA and Sham groups; *n* = 6. CFA: Complete Freund’s adjuvant.

### 3.2 Emodin effectively alleviates CFA-induced inflammatory pain

To evaluate the analgesic effect of emodin on inflammatory pain, mice were treated intraperitoneally with emodin at different doses (0, 5, 25, 50, or 100 mg/kg, once daily for five consecutive days) starting 3 days after CFA injection. Mechanical and thermal pain thresholds were assessed before CFA injection and after 5 days of emodin treatment (Figure 2A). The results showed that, compared with the pre-injection baseline, CFA significantly decreased pain thresholds and induced pronounced mechanical allodynia and thermal hyperalgesia in mice (Figures 2B,C). Intraperitoneal administration of emodin at 25 mg/kg significantly attenuated CFA-induced mechanical and thermal hypersensitivity. Moreover, emodin improved CFA-induced hyperalgesia in a dose-dependent manner, with the strongest analgesic effect observed at 50 mg/kg (Figures 2B,C).

**FIGURE 2:**
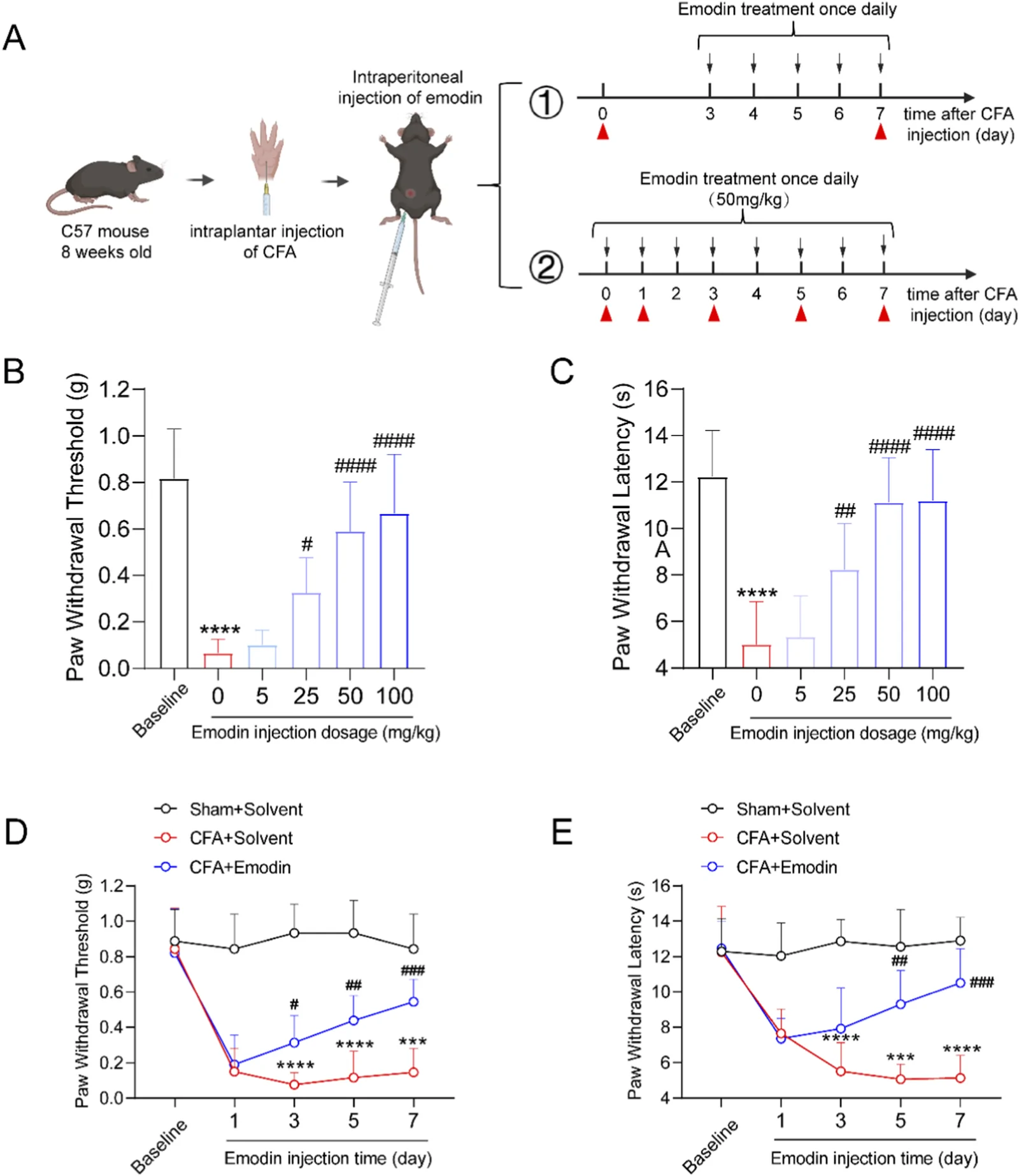
Emodin alleviates CFA-induced inflammatory pain in a dose-dependent manner. **(A)** Schematic diagram of the inflammatory pain mouse model and emodin treatment, including: 1) the administration regimen of emodin for the treatment of inflammatory pain; 2) the administration regimen of emodin for the prevention of inflammatory pain. **(B)** Von Frey test to evaluate the analgesic effect of emodin at different doses in inflammatory pain mice. **(C)** Hot plate test to evaluate the analgesic effect of emodin at different doses in inflammatory pain mice. All data are presented as mean ± SEM. Statistical analysis was performed using one-way ANOVA. ****P < 0.0001 indicates a significant difference in pain threshold between the CFA group and the baseline group; #*P* < 0.05, ##*P* < 0.01, ####*P* < 0.0001 indicate significant differences in pain threshold between the emodin-treated group and the CFA group. **(D)** Von Frey test to evaluate the preventive effect of emodin (50 mg/kg, intraperitoneal injection, once daily for eight consecutive days) on inflammatory pain. **(E)** Hot plate test to evaluate the preventive effect of emodin (50 mg/kg, intraperitoneal injection, once daily for eight consecutive days) on inflammatory pain. All data are presented as mean ± SEM. Statistical analysis was performed using two-way repeated-measures analysis of variance (ANOVA) followed by Sidak multiple-comparisons test. ****P < 0.0001 indicates a significant difference in pain threshold between the Saline group and the CFA group; #*P* < 0.05, ##*P* < 0.01, ###*P* < 0.001, ####*P* < 0.0001 indicate significant differences in pain threshold between the CFA + emodin group and the CFA group; n = 8. CFA, complete Freund’s adjuvant.

To further assess the preventive effect of emodin on inflammatory pain, mice received intraperitoneal emodin (50 mg/kg) as a prophylactic treatment on the day of CFA injection, once daily for eight consecutive days. Mechanical and thermal pain thresholds were measured before CFA injection and at 1, 3, 5, and 7 days after CFA injection (Figure 2A). Compared with the saline group (black), CFA injection markedly induced mechanical allodynia and thermal hyperalgesia in mice (Figures 2D,E, red). Prophylactic treatment with emodin (50 mg/kg) effectively alleviated CFA-induced inflammatory pain by day 4 of emodin administration, and the analgesic effect reached its maximum after 8 days of continuous treatment (Figures 2D,E, blue). Overall, these data indicate that emodin has substantial potential for both the prevention and treatment of CFA-induced inflammatory pain.

### 3.3 DEGs and biological functions in DRG tissues from CFA-induced inflammatory pain mice

Transcriptome sequencing was performed on L4–L6 DRG tissues isolated from mice with CFA-induced inflammatory pain. The DEG volcano plot (Figure 3A) identified a total of 136 differentially expressed genes, including 36 upregulated and 100 downregulated genes. The heatmap of the top 30 DEGs is shown in Figure 3B and includes pain-associated genes such as *Cx3cr1*.

**FIGURE 3:**
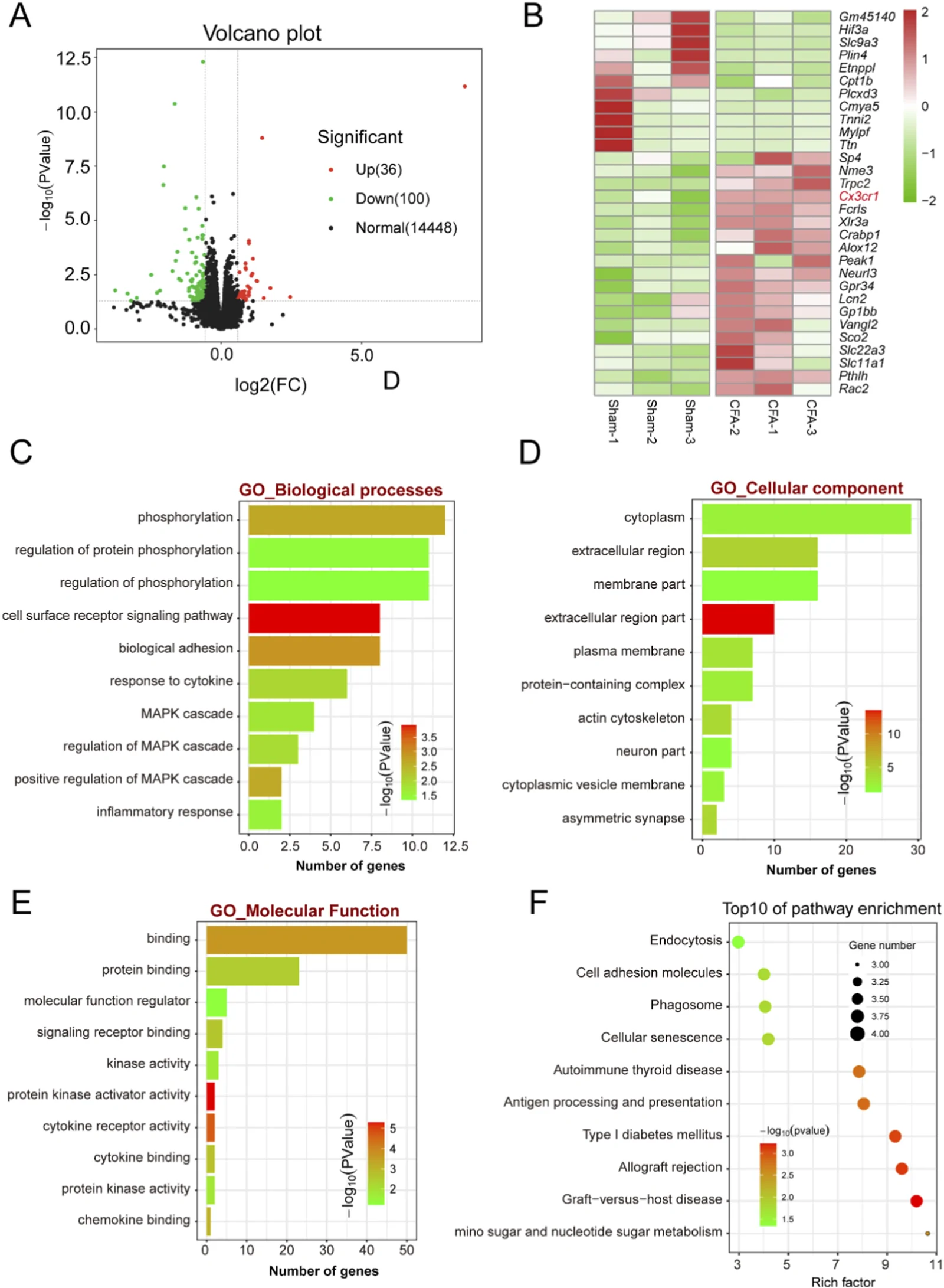
GO and KEGG enrichment analyses of DEGs in DRGs of mice with CFA-induced inflammatory pain. **(A)** Volcano plot of DEGs in DRGs of CFA-treated mice; **(B)** Heatmap showing expression profiles of the top 30 DEGs; **(C)** Enriched biological process (BP) terms of DEGs; **(D)** Cellular component (CC) terms; **(E)** Molecular function (MF) terms; **(F)** Enriched KEGG signaling pathways of DEGs.

GO enrichment analysis was then conducted on DEGs identified in the DRGs of CFA-injured mice. As shown in Figure 3C, the most significantly enriched biological process terms included response to cytokine, inflammatory response, cell surface receptor signaling pathway, MAPK cascade, and regulation of MAPK cascade. These findings suggest that the DEGs in mouse DRGs after CFA challenge are mainly involved in immune and inflammatory responses, and that the MAPK cascade may function as a downstream signaling pathway contributing to the progression of inflammatory pain. GO enrichment analysis for cellular component terms (Figure 3D) revealed that the DEGs were predominantly associated with cytoplasm, membrane part, plasma membrane, and protein-containing complex, indicating that these genes mainly exert their biological functions in the plasma membrane and cytoplasm. For molecular function terms (Figure 3E), the top enriched categories included binding, protein binding, kinase activity, cytokine receptor activity, and cytokine binding, suggesting that these DEGs are primarily involved in cytokine recognition and the activation of kinase-mediated signaling cascades.

KEGG pathway enrichment analysis of DEGs from the DRGs of CFA-treated mice (Figure 3F) highlighted antigen processing and presentation, cellular senescence, cell adhesion molecules, and endocytosis. These results indicate that DEGs in CFA-stimulated DRGs are mainly involved in antigen-related immune responses, as well as downstream processes such as cell adhesion, endocytosis, and cellular senescence.

### 3.4 Emodin downregulates Cx3cl1/Cx3cr1 expression in the DRG of CFA-induced inflammatory pain mice

To further determine the effect of emodin on Cx3cl1/Cx3cr1 expression in the DRG of CFA-induced inflammatory pain mice, emodin (25 or 50 mg/kg) was administered intraperitoneally once daily starting on the day of CFA injection for eight consecutive days, after which the DRG tissues were collected to assess Cx3cl1/Cx3cr1 mRNA and protein levels (Figure 4A). RT-qPCR analysis showed that the mRNA levels of Cx3cl1 and Cx3cr1 were significantly upregulated in the DRG tissues of CFA-induced inflammatory pain mice (Figures 4B,C). Emodin treatment at 25 or 50 mg/kg significantly suppressed the CFA-induced increases in *Cx3cl1* and *Cx3cr1* mRNA levels in the DRG (Figures 4B,C). Western blot analysis further revealed that Cx3cl1 and Cx3cr1 protein levels were markedly elevated in the DRG of CFA-treated mice, whereas emodin treatment at 50 mg/kg significantly reduced the protein expression of Cx3cl1 and Cx3cr1 in the DRG of CFA-treated mice (Figures 4D,E). In addition, immunofluorescence staining showed abnormally increased Cx3cr1-positive signals in the DRG of CFA-treated mice, which were significantly reduced following emodin treatment at 50 mg/kg (Figures 4F,G). Collectively, these results indicate that emodin suppresses the aberrant upregulation of Cx3cl1/Cx3cr1 in the DRG of CFA-induced inflammatory pain mice.

**FIGURE 4:**
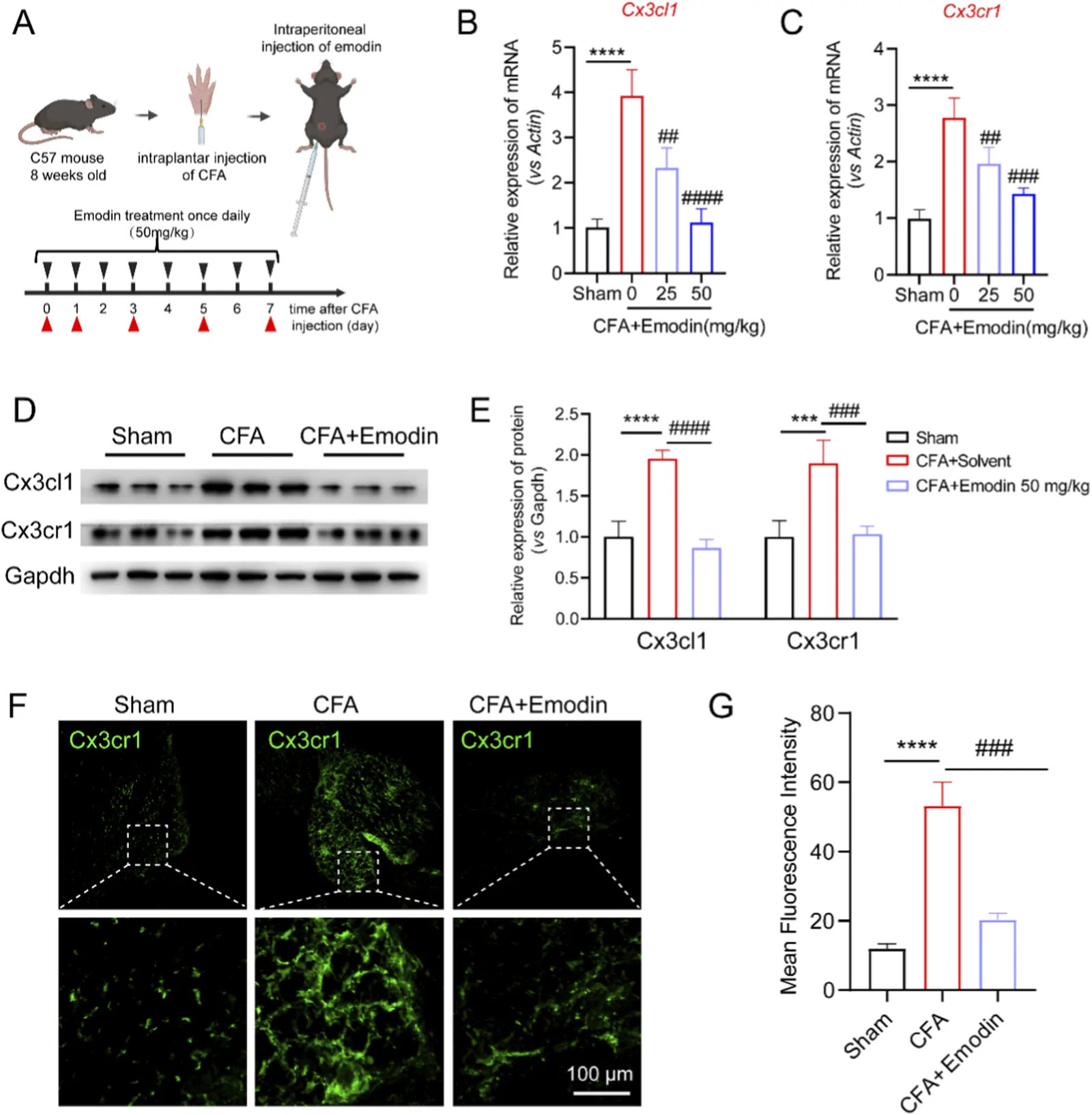
Emodin reverses the CFA-induced upregulation of Cx3cl1 and Cx3cr1. **(A)** Schematic diagram of the establishment of the CFA-induced inflammatory pain mouse model and emodin treatment (50 mg/kg, intraperitoneal injection, once daily for eight consecutive days). **(B,C)** RT-qPCR analysis of the mRNA expression levels of *Cx3cl1* and *Cx3cr1* in the DRG of mice after CFA injection and after emodin treatment. **(D)** Western blot analysis of Cx3cl1 and Cx3cr1 protein expression in the DRG of mice after CFA injection and after emodin treatment. **(E)** Quantitative analysis of Cx3cl1 and Cx3cr1 protein levels in the mouse DRG. **(F)** Immunofluorescence staining was used to detect Cx3cr1 protein expression in DRG tissue. **(G)** Quantitative analysis of Cx3cr1 immunofluorescence intensity. All data are presented as mean ± SEM. Statistical analysis was performed using one-way ANOVA. ***P < 0.001 and ****P < 0.0001 indicate significant differences between the Sham and CFA groups. ##*P* < 0.01, ###*P* < 0.001, and ####*P* < 0.0001 indicate significant differences between the CFA and CFA + emodin groups. Sham, control group; CFA, complete Freund’s adjuvant, *n* = 3.

### 3.5 Blocking Cx3cr1 effectively alleviates CFA-induced inflammatory pain

The marked upregulation of Cx3cl1 and Cx3cr1 in the DRG of CFA-treated mice suggests that the Cx3cl1/Cx3cr1 axis may be an important contributor to CFA-induced inflammatory pain. To clarify the role of the Cx3cl1/Cx3cr1 axis in CFA-induced inflammatory pain, mice received the Cx3cr1 inhibitor JMS-17-2 (1 or 10 mg/kg, once daily for seven consecutive days) on the day of CFA injection (Figure 5A), and mechanical and thermal pain thresholds were measured before CFA injection and on days 1, 3, 5, and 7 after JMS-17-2 administration. The results showed that, compared with the Sham + Solvent group, the CFA + Solvent group exhibited a significant decrease in mechanical and thermal pain thresholds beginning 1 day after CFA injection, and this reduction persisted until day 7 (Figures 5B,C, red). After intraperitoneal administration of the Cx3cr1 inhibitor JMS-17-2, mechanical and thermal pain thresholds in CFA-induced inflammatory pain mice were significantly increased in a dose-dependent manner (Figures 5B,C, green). In addition, 7 days after CFA injection, the effect of Cx3cr1 blockade on mechanical and thermal pain thresholds in CFA-induced inflammatory pain mice was evaluated by intrathecal injection of a Cx3cr1-specific antibody (1 or 10 μg) (Figure 5D). The results showed that, compared with the control IgG group, intrathecal administration of the Cx3cr1-specific antibody significantly increased the mechanical and thermal pain thresholds of CFA-treated mice in a dose-dependent manner, with the maximal effect observed 3 h after injection (Figures 5E,F). Intrathecal injection of a high dose of the Cx3cr1-specific antibody (10 μg) maintained the relief of mechanical allodynia and thermal hyperalgesia for 6 h, whereas pain thresholds decreased to the level of the IgG group 24 h after injection (Figures 5E,F).

**FIGURE 5:**
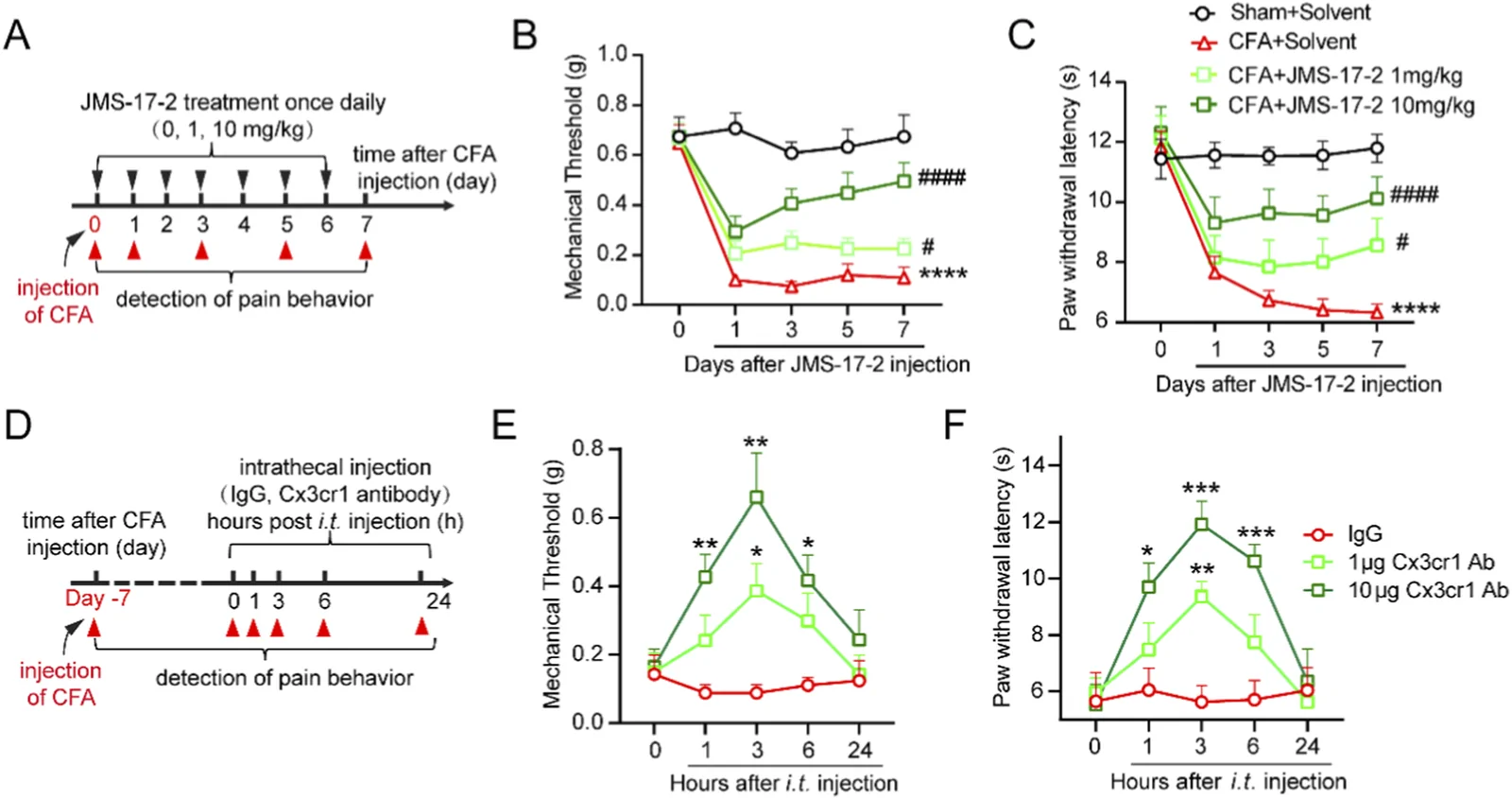
Blockade of Cx3cr1 effectively relieves CFA-induced pain hyperalgesia. **(A)** Schematic diagram of the treatment of CFA-induced inflammatory pain with the Cx3cr1 antagonist JMS-17-2 (once daily for seven consecutive days). **(B)** Von Frey test assessing the effect of JMS-17-2 treatment on the mechanical pain threshold in CFA mice, *n* = 8. **(C)** Hot plate test assessing the effect of JMS-17-2 treatment on the thermal pain threshold in CFA mice, *n* = 8. All data are presented as mean ± SEM. Statistical analysis was performed using two-way repeated-measures analysis of variance (ANOVA) followed by Sidak multiple-comparisons test. ****P < 0.0001 indicates a significant difference in pain threshold between the Sham + Solvent group and the CFA + Solvent group; #*P* < 0.05 and ####*P* < 0.0001 indicate significant differences in pain threshold between the CFA + Solvent group and the CFA + JMS-17-2 group. **(D)** Schematic diagram of CFA-induced inflammatory pain treatment with a Cx3cr1-specific antibody or control IgG administered once *via* intrathecal injection. **(E)** Von Frey test assessing the effect of Cx3cr1 antibody treatment on the mechanical pain threshold in CFA mice, *n* = 8. **(F)** Hot plate test assessing the effect of Cx3cr1 antibody treatment on the thermal pain threshold in CFA mice, *n* = 8. All data are presented as mean ± SEM. Statistical analysis was performed using two-way repeated-measures analysis of variance (ANOVA) followed by Sidak multiple-comparisons test. *P < 0.05, **P < 0.01 and ***P < 0.001 indicate significant differences in pain threshold between the CFA + IgG group and the CFA + Cx3cr1 Ab group. CFA, complete Freund’s adjuvant; JMS-17-2, Cx3cr1 antagonist; IgG, control antibody.

To further define the role of Cx3cr1 in CFA-induced inflammatory pain, AAV shRNA targeting Cx3cr1 was intrathecally injected into mice 2 weeks before CFA injection, and mechanical and thermal pain thresholds were dynamically assessed for 28 days after AAV administration (Figure 6A). The results showed that intrathecal injection of AAV shRNA targeting Cx3cr1 significantly reduced Cx3cr1 mRNA and protein levels in the DRG of CFA-treated mice (Figures 6B–D). Meanwhile, Cx3cr1 knockdown markedly alleviated CFA-induced mechanical allodynia and thermal hyperalgesia (Figures 6E,F). These findings suggest that Cx3cr1 may be involved in the regulation of inflammatory pain and may contribute to the analgesic effects of emodin.

**FIGURE 6:**
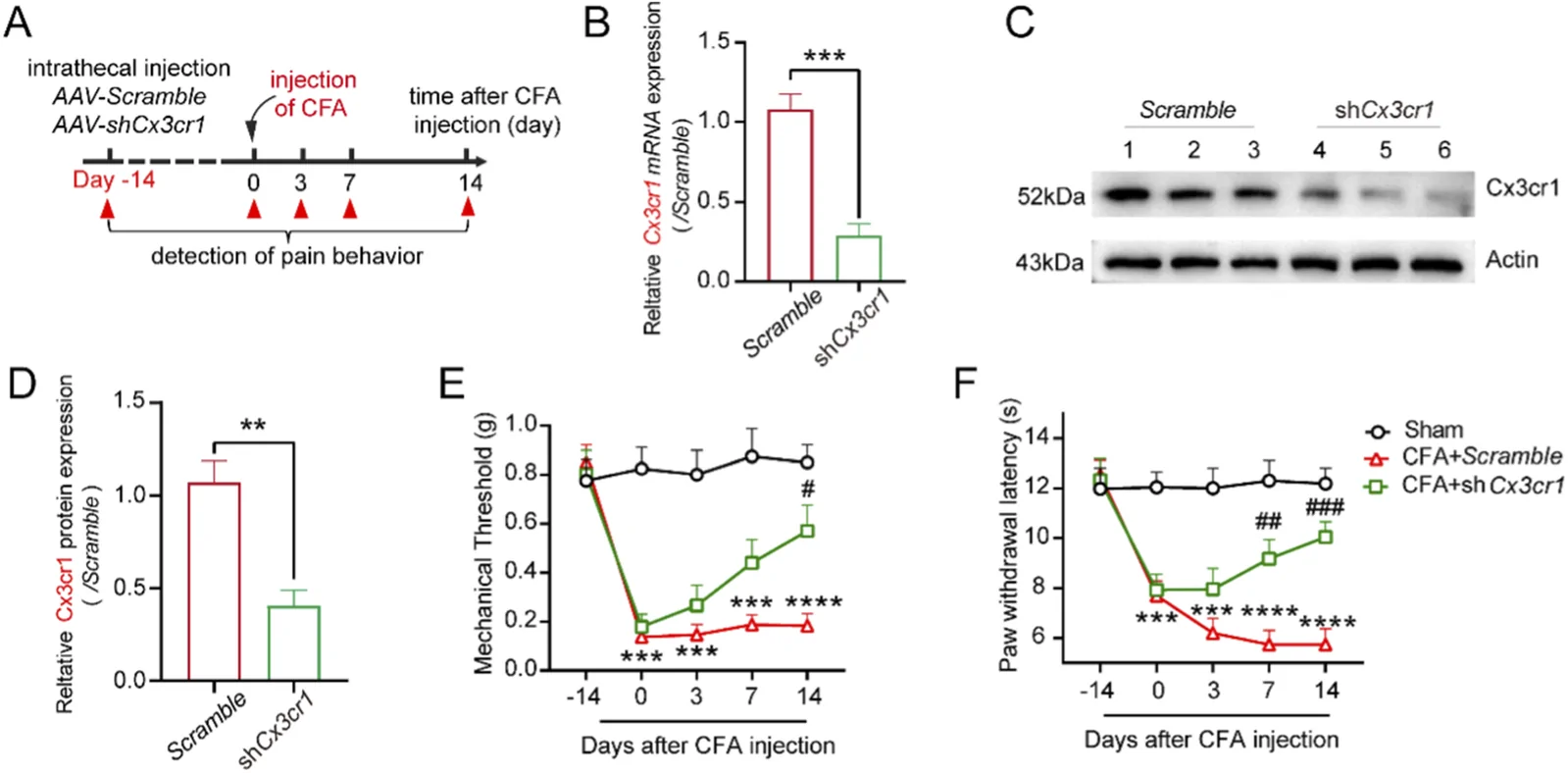
Targeted knockdown of Cx3cr1 alleviates CFA-induced inflammatory pain. **(A)** Schematic diagram of CFA-induced inflammatory pain treatment via intrathecal injection of adeno-associated virus carrying Cx3cr1-targeting shRNA or a scrambled sequence. **(B)** RT-qPCR analysis of *Cx3cr1* mRNA expression levels in the DRG. **(C)** Western blot analysis of Cx3cr1 and actin protein expression in the DRG of mice. **(D)** Quantitative analysis of Cx3cr1 protein levels in the mouse DRG. All data are presented as mean ± SEM. Statistical analysis was performed using one-way ANOVA. **P < 0.01 and ***P < 0.001 indicate significant differences in Cx3cr1 levels between the Cx3cr1 knockdown group and the Scramble group. **(E)** Von Frey test assessing the effect of Cx3cr1 knockdown on the mechanical pain threshold in CFA mice, *n* = 8. **(F)** Hot plate test assessing the effect of Cx3cr1 knockdown on the thermal pain threshold in CFA mice, *n* = 8. All data are presented as mean ± SEM. Statistical analysis was performed using two-way repeated-measures analysis of variance (ANOVA) followed by Sidak multiple-comparisons test. ***P < 0.001 and ****P < 0.0001 indicates a significant difference in pain threshold between the Sham group and the CFA + Scramble group; #*P* < 0.05, ##*P* < 0.01 and ###*P* < 0.001 indicate significant differences in pain threshold between the CFA + Scramble group and the CFA + shCx3cr1 group. CFA, complete Freund’s adjuvant; AAV, adeno-associated virus; Scramble, scrambled sequence.

### 3.6 Emodin suppresses the immune-inflammatory response in the DRG of CFA-treated mice by regulating the Cx3cl1/Cx3cr1/p38 MAPK signaling pathway

To further investigate whether emodin may interact with Cx3cr1 and thereby modulate its biological activity to exert analgesic effects, molecular docking analysis was performed between emodin and the Cx3cr1 protein (Figure 7, left). The results showed that GLY176, GLY178, and TYR190 of Cx3cr1 formed hydrogen bonds with emodin; PRO189, PHE111, and TRP88 formed hydrophobic interactions; THR169, LEU177, THR107, ASN193, GLU255, and PHE259 formed van der Waals interactions; and ARG192 formed a pi-cation interaction with emodin (Figure 7, right). The predicted binding energy between Cx3cr1 and emodin was −7.2 kcal/mol, suggesting a potentially favorable binding interaction. Docking analysis further predicted a plausible binding mode between Cx3cr1 and emodin.

**FIGURE 7:**
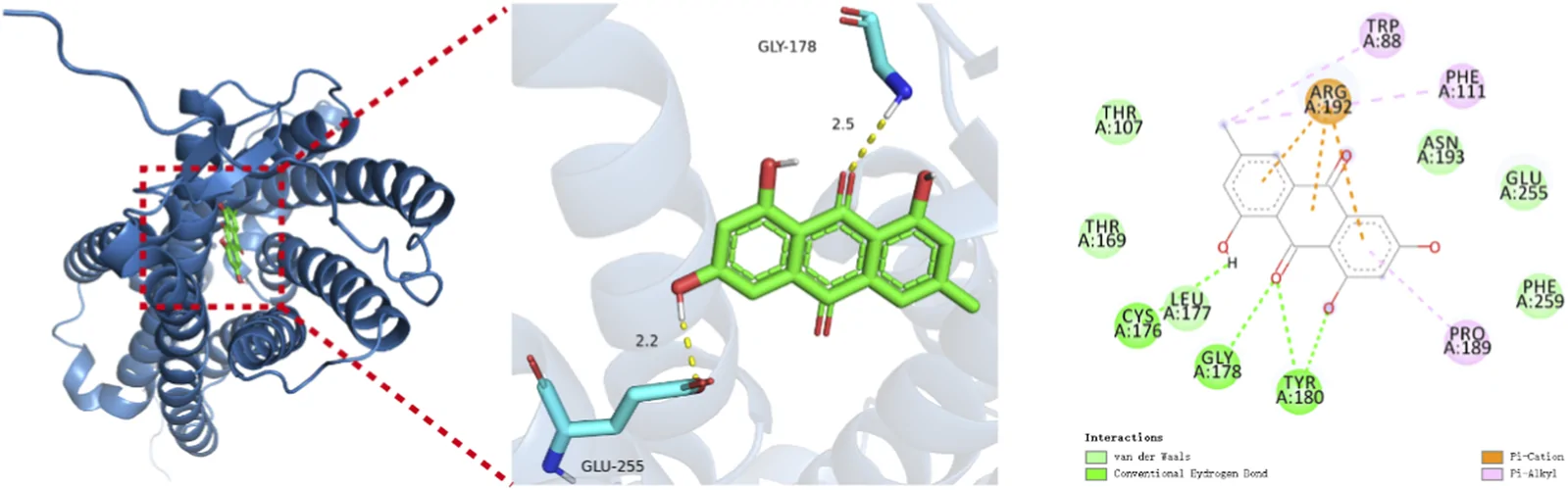
Molecular docking of emodin with Cx3cr1. The GLY176, GLY178, and TYR190 residues of the Cx3cr1 receptor formed hydrogen bond interactions with emodin. The PRO189, PHE111, and TRP88 residues formed hydrophobic interactions with emodin. The THR169, LEU177, THR107, ASN193, GLU255, and PHE259 residues formed van der Waals interactions with emodin. The ARG192 residue formed a pi-cation interaction with emodin. The binding energy of the Cx3cr1-emodin complex was −7.2 kcal/mol, indicating strong binding affinity between the two molecules.

To elucidate the downstream mechanism by which emodin alleviates CFA-induced inflammatory pain, emodin (50 mg/kg) was administered intraperitoneally once daily for eight consecutive days starting on the day of CFA injection, after which DRG tissues were collected for H&E staining, Western blotting, and RT-qPCR analyses. Histopathological examination of H&E-stained DRG sections revealed obvious immune-inflammatory infiltration in the DRG of the CFA + Solvent group compared with the Sham + Solvent group (Figure 8A, middle), whereas emodin treatment effectively reduced immune cell infiltration in the DRG (Figure 8A, right). Western blot analysis showed that the protein levels of Cx3cl1, Cx3cr1, and p-p38/p38 were significantly increased in the DRG of CFA-induced inflammatory pain mice, and these CFA-induced increases were markedly reversed by emodin treatment (Figures 8B–E). RT-qPCR analysis of proinflammatory cytokine mRNA in the DRG demonstrated that the mRNA levels of *TNF-*α, *IL-1*β, and *IL-6* were abnormally elevated in CFA-induced inflammatory pain mice, whereas emodin significantly suppressed the expression of these proinflammatory cytokines in the DRG (Figures 8F–H).

**FIGURE 8:**
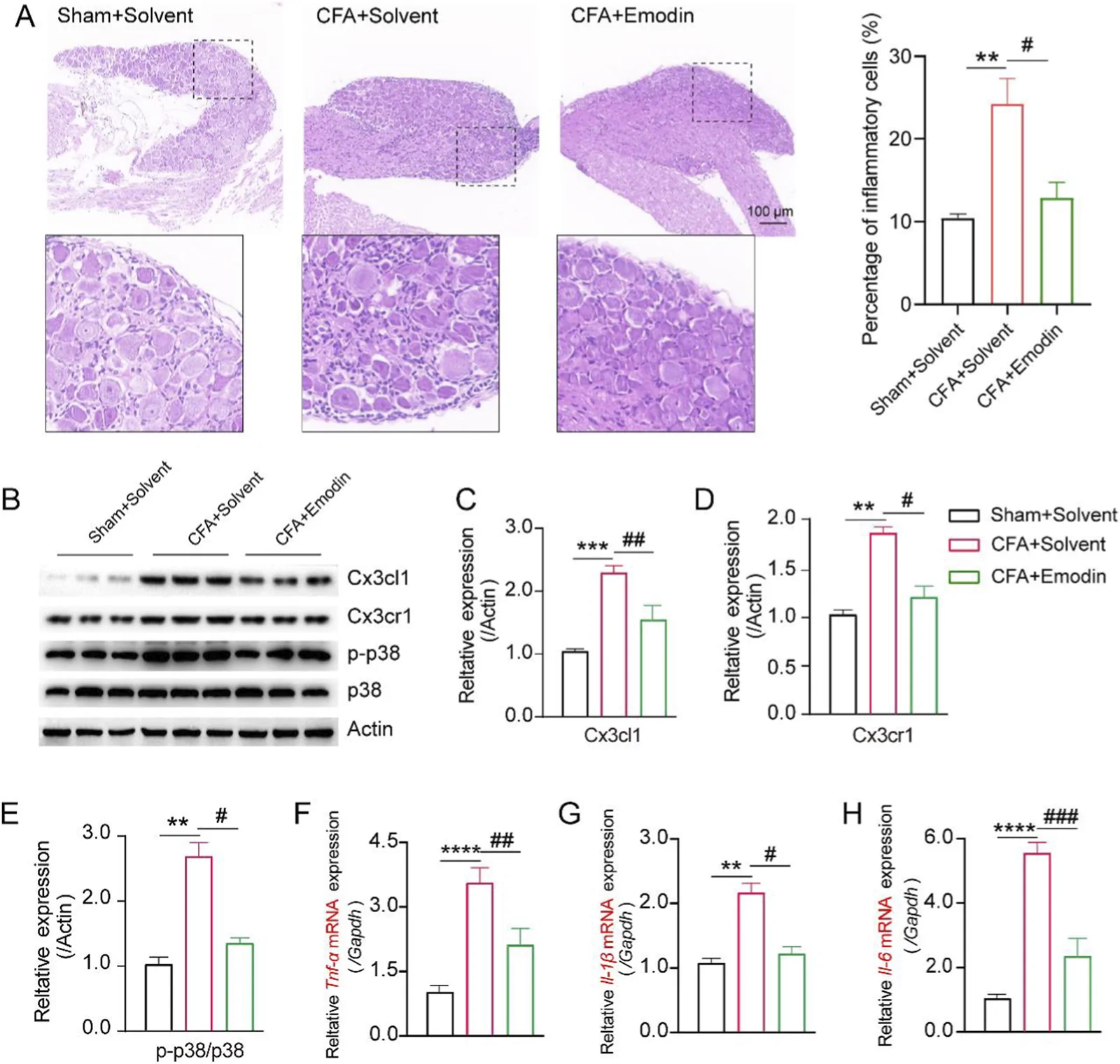
Emodin suppresses immune cell infiltration and neuroinflammation in the DRG of CFA-induced inflammatory pain mice by downregulating the Cx3cl1/Cx3cr1/p38 MAPK pathway. **(A)** Hematoxylin and eosin staining was used to assess immune cell infiltration in the DRG of mice in each group. Quantification of the percentage of inflammatory cells in the infiltrated area using Image J. **(B)** Western blot analysis of Cx3cl1, Cx3cr1, p-p38, p38, and actin protein levels in mouse DRG tissues. **(C–E)** Densitometric analysis of the Cx3cl1, Cx3cr1, and p-p38/p38 bands. **(F–H)** RT-qPCR analysis of the mRNA expression levels of the proinflammatory cytokines *Tnf-α* **(F)**, *Il-1β* **(G)**, and *Il-6* **(H)** in the mouse DRG. All data are presented as mean ± SEM. Statistical analysis was performed using one-way ANOVA, *n* = 3. **P < 0.01, ***P < 0.001, and ****P < 0.0001 indicate significant differences between the Sham group and the CFA group. #*P* < 0.05, ##*P* < 0.01, and ###*P* < 0.001 indicate significant differences between the CFA group and the CFA + emodin group.

These findings suggest that emodin may alleviate CFA-induced inflammatory pain in association with modulation of the Cx3cl1/Cx3cr1/p38 MAPK signaling axis in the DRG, accompanied by reduced immune-cell infiltration and decreased production of proinflammatory cytokines.

## 4 Discussion

Inflammatory pain remains a major clinical challenge because peripheral tissue inflammation can trigger persistent nociceptive sensitization and long-lasting pain hypersensitivity (Yang et al., 2023). In the present study, CFA injection induced robust mechanical allodynia and thermal hyperalgesia in mice, confirming successful establishment of an inflammatory pain model (Figure 1). Both prophylactic and therapeutic administration of emodin significantly alleviated these pain behaviors, indicating that emodin exerts not only preventive but also post-insult analgesic effects (Figure 2). Such findings are consistent with the growing recognition that natural anthraquinone compounds can modulate pain-related inflammatory pathways and nociceptive signaling, suggesting that emodin may represent a promising candidate for inflammatory pain intervention (Cheng et al., 2024; Cheng et al., 2022; Zhang et al., 2023).

A central finding of this study is that CFA-induced inflammatory pain was accompanied by marked transcriptomic remodeling in DRG tissue, with prominent enrichment of genes associated with neuroinflammation and immune activation. In particular, Cx3cr1 was significantly upregulated, and pathway analysis highlighted MAPK signaling as one of the most enriched pathways (Figures 3B,C). This is biologically meaningful because DRG neurons and resident immune-related cells serve as key interfaces between peripheral inflammation and central nociceptive transmission (Hao et al., 2023). Accumulating evidence indicates that transcriptomic changes in the DRG are not merely passive consequences of inflammation but active contributors to peripheral sensitization and pain chronicity (Hall et al., 2024; Yao et al., 2022). Therefore, the transcriptional signature observed here supports the concept that CFA induces a neuroimmune state in the DRG that promotes pain hypersensitivity.

CX3CL1 is a chemokine that mediates neuron-glia and neuron-immune communication through binding to its cognate receptor CX3CR1 (Old and Malcangio, 2012; Sheridan and Murphy, 2013). In pain-related contexts, this signaling pathway has been implicated in the recruitment and activation of immune cells, glial activation, and amplification of inflammatory nociception (Old and Malcangio, 2012). Our data showed that CFA markedly increased Cx3cl1/Cx3cr1 mRNA and protein levels in DRG tissue, whereas emodin treatment reversed these changes (Figure 4). Moreover, pharmacological and genetic blockade of CX3CR1 significantly relieved CFA-induced hyperalgesia, providing functional evidence that the Cx3cl1/Cx3cr1 signaling axis contributes directly to inflammatory pain generation (Figures 5, 6). These findings extend prior reports by placing the CX3CL1/CX3CR1 pair in the DRG as an important mediator of CFA-driven peripheral sensitization. Finally, the molecular docking results suggested that Cx3cr1 may be involved in the biological effects of emodin on inflammatory pain; however, this possibility requires further confirmation through biophysical binding assays and other independent validation approaches.

The MAPK pathway is another major mechanistic node supported by our transcriptomic data (Figure 3C). CFA increased phosphorylation of p38 in the DRG, and emodin substantially suppressed p-p38/p38 expression (Figure 8B). MAPK cascades, particularly p38 MAPK, are widely recognized as key signaling modules that translate inflammatory cues into altered neuronal excitability, cytokine production, and immune-cell activation (Alam et al., 2018; Cadenas-Garrido et al., 2026; Sun et al., 2025). In sensory ganglia, p38 activation has been linked to enhanced expression of inflammatory mediators and maintenance of hyperalgesia (Mizushima et al., 2005). Thus, the inhibitory effect of emodin on p38 phosphorylation suggests that the compound may suppress a central downstream signaling route required for inflammatory pain amplification. Importantly, the convergence of receptor-level regulation, and functional blockade experiments supports a model in which emodin suppresses inflammatory pain through inhibition of the Cx3cl1/Cx3cr1/p38 MAPK axis rather than through a single isolated target. Although molecular docking analysis suggested that emodin may interact with Cx3cr1, this possibility remains hypothetical and requires validation using direct molecular-binding and other mechanistic approaches.

Another important observation is that emodin reduced immune cell infiltration in DRG tissue and lowered the expression of the proinflammatory cytokines *TNF-*α, *IL-1*β, and *IL-6* (Figure 8). These cytokines are established mediators of pain sensitization because they can directly enhance nociceptor responsiveness, alter ion channel activity, and promote a feed-forward inflammatory loop within sensory ganglia (Barker et al., 2022; Goncalves Dos Santos et al., 2019). The reduction in cytokine expression after emodin treatment is therefore highly relevant to its analgesic action. Together, these findings support the view that emodin does not merely suppress nociceptive behavior symptomatically but attenuates the inflammatory microenvironment that sustains pain signaling in the DRG.

Mechanistically, the present study suggests that emodin may interrupt a neuroimmune cascade initiated by CFA-induced inflammation. One plausible model is that CFA triggers chemokine signaling in the DRG, leading to activation of CX3CR1-expressing cells and downstream p38 MAPK signaling, which in turn drives cytokine production and immune-cell recruitment. Emodin appears to interfere with this axis at multiple levels: it downregulates Cx3cl1/Cx3cr1 expression, inhibits p38 activation, reduces immune infiltration, and suppresses proinflammatory cytokines (Figure 8). Multi-level modulation of this kind is often characteristic of bioactive natural products and may partly explain why emodin showed efficacy in both preventive and therapeutic paradigms (Figure 2).

Increasing evidence indicates that neuroimmune communication within peripheral sensory ganglia is a critical contributor to the initiation and maintenance of inflammatory pain. In particular, activation of immune-related signaling pathways and the subsequent release of pro-inflammatory mediators in the DRG can sensitize primary sensory neurons and promote persistent nociceptive hypersensitivity (Chen et al., 2026). Recent studies have further emphasized the importance of inflammatory and neuroimmune mechanisms in pain modulation (Li et al., 2023; Li et al., 2025). Consistent with these findings, CFA administration in the present study was associated with increased immune cell infiltration and elevated expression of Tnf-α, Il-1β, and Il-6 in the DRG, accompanied by activation of the Cx3cl1/Cx3cr1/p38 MAPK signaling axis. Emodin treatment markedly attenuated these alterations, suggesting that its antinociceptive effect may be attributable, at least in part, to the suppression of DRG neuroinflammation and Cx3cl1/Cx3cr1/p38 MAPK-related signaling.

A limitation of the present study is that the cellular localization of Cx3cr1 within the DRG was not determined. Because the DRG contains heterogeneous cell populations, including sensory neurons, satellite glial cells, and macrophages, the changes in Cx3cr1 observed in the present study should be interpreted as whole-DRG alterations and cannot be assigned to a specific cell type. Consequently, our findings do not establish the primary cellular target through which emodin exerts its analgesic effects. Future studies should perform double- or multiple-immunofluorescence co-staining of Cx3cr1 with macrophage markers (e.g., Iba1 or F4/80), SGC markers (e.g., S100β or GS), and neuronal markers (e.g., NeuN), combined with cell type-specific functional approaches, to clarify the cellular basis of emodin-mediated analgesia.

## 5 Conclusion

In summary, emodin attenuated CFA-induced inflammatory pain in mice. This analgesic effect was associated with reduced Cx3cl1/Cx3cr1 signaling, decreased p38 MAPK activation, diminished immune-cell infiltration in the DRG, and downregulation of proinflammatory cytokines. These findings suggest that modulation of the DRG neuroimmune microenvironment may contribute to the analgesic effects of emodin and indicate that the Cx3cl1/Cx3cr1/p38 MAPK signaling axis may be involved in this process.

## Data Availability

The data presented in the study are deposited in the China National Center for Bioinformation (CNCB) repository, accession number PRJCA072250.
